# A Graphical User Interface for a Method to Infer Kinetics and Network Architecture (MIKANA)

**DOI:** 10.1371/journal.pone.0027534

**Published:** 2011-11-11

**Authors:** Márcio A. Mourão, Jeyaraman Srividhya, Patrick E. McSharry, Edmund J. Crampin, Santiago Schnell

**Affiliations:** 1 Department of Molecular and Integrative Physiology, University of Michigan Medical School, Ann Arbor, Michigan, United States of America; 2 The Biocomplexity Institute, Department of Physics, Indiana University, Bloomington, Indiana, United States of America; 3 Smith School of Enterprise and the Environment, University of Oxford, Oxford, United Kingdom; 4 Mathematical Institute, University of Oxford, Oxford, United Kingdom; 5 Auckland Bioengineering Institute, University of Auckland, Auckland, New Zealand; 6 Department of Engineering Science, University of Auckland, Auckland, New Zealand; 7 Center for Computational Medicine & Bioinformatics, University of Michigan Medical School, Ann Arbor, Michigan, United States of America; 8 Brehm Center for Diabetes Research, University of Michigan Medical School, Ann Arbor, Michigan, United States of America; University of Georgia, United States of America

## Abstract

One of the main challenges in the biomedical sciences is the determination of reaction mechanisms that constitute a biochemical pathway. During the last decades, advances have been made in building complex diagrams showing the static interactions of proteins. The challenge for systems biologists is to build realistic models of the dynamical behavior of reactants, intermediates and products. For this purpose, several methods have been recently proposed to deduce the reaction mechanisms or to estimate the kinetic parameters of the elementary reactions that constitute the pathway. One such method is MIKANA: **M**ethod to **I**nfer **K**inetics **A**nd **N**etwork **A**rchitecture. MIKANA is a computational method to infer both reaction mechanisms and estimate the kinetic parameters of biochemical pathways from time course data. To make it available to the scientific community, we developed a Graphical User Interface (GUI) for MIKANA. Among other features, the GUI validates and processes an input time course data, displays the inferred reactions, generates the differential equations for the chemical species in the pathway and plots the prediction curves on top of the input time course data. We also added a new feature to MIKANA that allows the user to exclude *a priori* known reactions from the inferred mechanism. This addition improves the performance of the method. In this article, we illustrate the GUI for MIKANA with three examples: an irreversible Michaelis–Menten reaction mechanism; the interaction map of chemical species of the muscle glycolytic pathway; and the glycolytic pathway of *Lactococcus lactis*. We also describe the code and methods in sufficient detail to allow researchers to further develop the code or reproduce the experiments described. The code for MIKANA is open source, free for academic and non-academic use and is available for download (Information S1).

## Introduction

The biological revolution unleashed by the use of high–throughput technologies provides a detailed picture of the protein interaction map of diverse organisms. The investigation of complex protein interaction maps requires the development of methodologies to understand the complex associations, localization and binding partners among thousands of proteins [1]–[4]. These methodologies provide an effective static picture of protein function in a biochemical pathway. Now the challenge for systems biologists is in building realistic models of the dynamical behavior by reconstructing the reaction mechanisms of proteins [5], [6].

For more than a century, the traditional experimental practice to investigate reaction mechanisms and estimate reaction kinetic parameters has entailed measuring the reactants and products as a function of time [7], [8]. Textbooks in chemical and enzyme kinetics illustrate diverse approaches for the estimation of kinetic parameters and determination of reaction mechanisms [9]–[11]. During the last 40 years, computational tools have emerged to model biochemical pathways and to estimate kinetic parameters [12]. However, these computational tools require detailed knowledge of the reaction mechanisms to estimate kinetic parameters or deduce specific mechanistic behaviors. More recently, several approaches have been proposed for the reconstruction of reaction mechanisms based on the causal chemical connectivity of the species [13], [14], singular value decomposition of the reaction velocities [15] and on the sequence of elementary reactions among that constitute the reaction mechanisms [16]–[18]. We have extensively reviewed these and other approaches for reconstructing mechanisms of biochemical pathways in [6].

MIKANA (**M**ethod to **I**nfer **K**inetics **A**nd **N**etwork **A**rchitecture) is a computational method to infer reaction mechanisms and estimate the kinetic parameters of biochemical pathways from time course data. It identifies the sequence of elementary reaction steps of a biochemical pathway using a global nonlinear modeling technique. The method involves the generation of a complete dictionary of polynomial basis functions based on the law of mass action. MIKANA is described in Srividhya et al. [17]. The sequential reconstruction of elementary reactions is guided by a cost function, an Information Criterion that penalizes the use of an excessive number of reactions to reconstruct the mechanism. The effectiveness of MIKANA to reconstruct the reaction mechanisms and estimate kinetic parameters relies on the experimental design employed on the measurements of the concentration of chemical species [19]. For enzyme catalyzed reactions, the sensitivity of the method varies with initial substrate–enzyme concentration ratios, initial substrate–enzyme concentration variation ranges, number of data points, number of different experiments (time courses) and experimental noise. We have also found that MIKANA's accuracy in the reconstruction of the reaction topology depends on the number of chemical species and their reaction kinetics. MIKANA can reconstruct accurately the elementary reaction mechanisms of eight interacting species following first and second order kinetics. The reconstruction has also shown to be effective for mechanisms with a larger number of interacting species, as long as the mechanisms are accurately described with pseudo-first order kinetics [19], [20].

In this paper, we present a Graphical User Interface (GUI) for MIKANA. Users can employ the GUI to input, validate, process and interpret the results of the reconstruction of a mechanism. We also added a new feature to MIKANA, which permits the exclusion of physically or biochemically unrealistic reactions from the inferred mechanism. This addition improves the performance of the method. We illustrate the GUI with three practical examples, described in the results section. Importantly, this article provides a detailed description of the methods and source code of MIKANA, so that any researcher can reproduce the experiments and/or continue the development of the software.

## Results

In MIKANA, the task of identifying biochemical pathways from time course data consists of, firstly, identifying the connectivity of the pathway (the reaction diagram relating reactants and products) and, secondly, determining and parameterizing the reaction mechanisms involved in each step of the pathway. This requires a good deal of chemical knowledge about plausible interconversions for the species in the pathway. We managed to incorporate this chemical knowledge by reconstructing reaction mechanisms from time course data using a global nonlinear modeling technique to identify the elementary reaction steps which constitute the pathway. The identification is made by selecting reactions from a dictionary of functions, which is built by assuming mass action kinetics in all chemically plausible steps between the chemical species measured in the whole reaction system. For a description of MIKANA, we invite the user to read [17]. We built a Graphical User Interface (GUI) for MIKANA, which we describe below.

### MIKANA's Graphical User Interface

**Figure 1:**
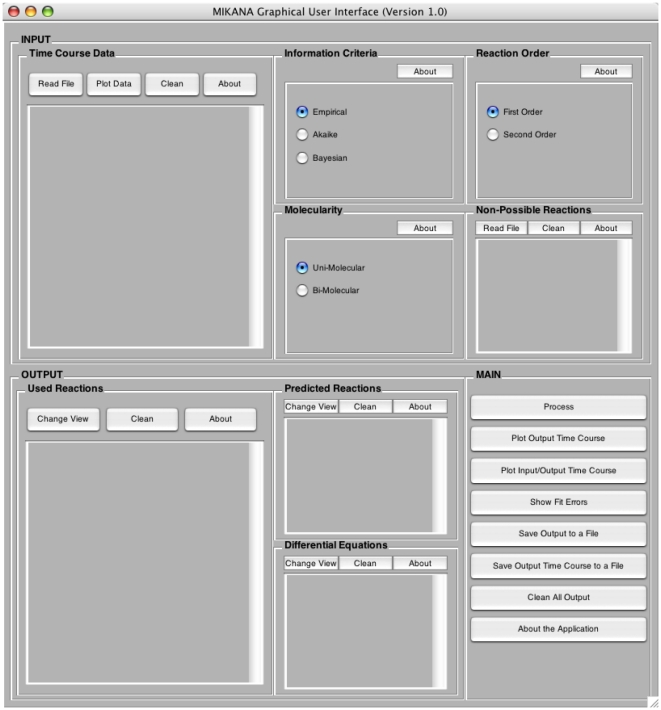
MIKANA's Graphical User Interface (GUI). The GUI is composed of three main panels: (i) the INPUT panel (at the top); (ii) the OUTPUT panel (at the bottom left); and (iii) the MAIN panel (bottom right).

The MIKANA's GUI is composed of three separate panels: (i) the INPUT panel (at the top); (ii) the OUTPUT panel (at the bottom left); and (iii) the MAIN panel (bottom right) (Figure 1). The Input is composed of five subpanels. At the left, the Time Course Data subpanel is used to read the input time course data from a file.

The input time course data file should contain a header or first line with the chemical species names or symbols. The interface accepts as many symbols as the number of chemical species. The following lines should only contain numeric data or an invalid insertion will be reported back to the user. In the numeric data, each line should contain one time column plus n chemical species columns. The time column should contain the time at which the following chemical species values associate, as in Table 1. Importantly, all columns should contain the same number of data points. In order to obtain plausible reaction steps from MIKANA, the time series should also be reduced to only contain values that change over the time period selected. This occurs because MIKANA bases the inference of the chemical species network and kinetics rate on the dynamics of the network, as reflected by the change on the concentration of the chemical species over time. Failure to address this issue may result in inaccurate predictions of the network and kinetics rate.

**Table 1:** Time course data file example.

	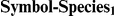	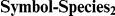	...	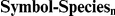
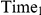	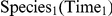	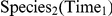	...	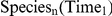
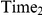	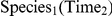	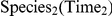	...	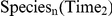
...	...	...	...	...
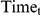	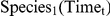	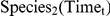	...	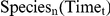

The first column of the table contains the time index. All other columns contain the chemical species concentration at the time referred to in the first column.

After successfully reading a time course data file, an option to plot and visualize the data over time is made available through the button Plot Data. The other four Input subpanels are made available as options to select an information criterion (Empirical, Akaike or Bayesian), a reaction order (First order or Second order), interaction molecularity (uni– or bi–molecular) and a set of reactions to exclude from the final reaction set solution. These latter reactions are read from a file and follow the grammar described previously. We generate six different types of error messages based on different types of errors that may arise upon insertion of a reactions set file:

- Reactions file not found or permission denied
- Number of molecules not allowed in reaction
- Species identifier greater than number of species available
- Reaction symbol 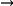 expected
- Character not allowed
- Empty file

To process the data and infer the metabolic pathway, the user will need to click the button Process (the first button in the Main panel). The Output panel (bottom left) of the GUI is composed of three subpanels. At the left, the Used reactions subpanel shows all elementary reactions considered in the model selection process. The Predicted reactions subpanel shows the final selection of reactions that constitute the inferred metabolic pathway. The Differential equations subpanel shows a set of differential equations that correspond to the final mechanism. We provide three different views of the results in each of these subpanels. The first view displays all chemical species that participate in the reactions. The second view excludes from the reactions species with zero stoichiometry. The third and last view display the chemical species by their name or symbol, as introduced in the input time course data file.

**Figure 2:**
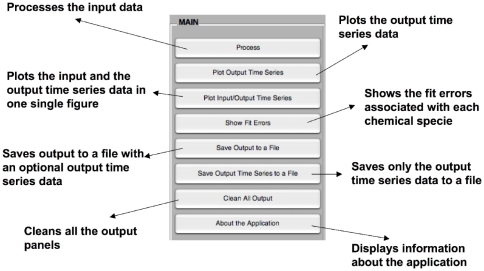
MIKANA's GUI Main Panel functions. The Main panel (bottom right) of the GUI is composed of eight buttons.

The Main panel (bottom right) of the GUI is composed of eight buttons (Figure 2). The first button (Process) processes the input data. The second button (Plot Output Time Course) plots the chemical species concentrations versus time from the inferred differential equations and kinetic rate constants. The third button (Plot Input/Output Time Course) plots the input and the output time course data in the same figure. The fourth button (Show Fit Errors) shows the fit error associated with each one of the chemical species. The fifth and sixth buttons allow the user to save all the results (Save Output to a File), or just the output time course data to a file (Save Output Time Course to a File), respectively. When saving results to a file, the used, predicted and differential equations are saved according to the current selected output view in these subpanels. These saving options could be useful to provide results that can be used in subsequent work. The last two buttons (Clean All Output and About the Application) cleans all the information provided in the output panel of the GUI and provides information about the application, respectively.

### Examples of reconstruction of reaction mechanisms using the MIKANA GUI

To illustrate the GUI for MIKANA, we reconstructed the reaction mechanisms of three biochemical pathways. The first case deals with one of the simplest pathways in biochemistry: the single-enzyme, single-substrate catalyzed reaction following a Michaelis-Menten mechanism. In this example, we used ‘in silico’ time course data, which was analyzed using MIKANA in a previous paper [19]. In a second example, we reconstructed the interaction map of three chemical species of the muscle glycolytic cell pathway. The experimental time course data from which we extracted the data was originally obtained by Scopes [21], [22]. The third example deals with a more complex pathway: the temporal evolution of seven species of the glycolytic pathway in *Lactococcus lactis*. This pathway was also analyzed using MIKANA in a previous paper [17].

### Example 1 - The irreversible Michaelis–Menten reaction mechanism

As a practical example, we considered the single-enzyme, single-substrate reaction, known as the Michaelis–Menten (MM) mechanism of enzyme action [11]. The reaction scheme is represented schematically by
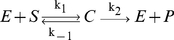
(1)Here, 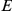 is the enzyme, 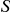 is the substrate and 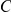 is the enzyme-substrate intermediate complex. Applying the law of mass action, we wrote the ordinary differential equation system describing the above reaction,
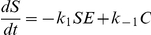


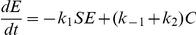


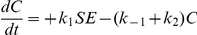


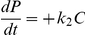



and solved it numerically in MATLAB with the stiff solver ‘ode23s.m’.

We selected the kinetic parameters from reported literature in the hydrolysis of Dnp-ADCA (7-(2′, 4′-dinitrophenylamino) deacetoxycephalosporanic acid) by 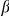-lactamase I from *Bacillus cereus* [23]. These parameters are: 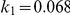 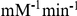, 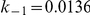 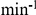 and 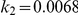 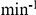. This produced time courses for four species: the substrate (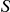), the enzyme (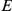), the complex (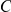) and the product (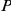). We added 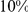 noise to the time course data to mimic noise levels observed in experimental data.

For the MM system, the header line contains 4 symbols and the numeric data consists of 30 lines and five columns. Along with the MIKANA package, we provide an example input file for the MM system. To read it, the user should click the button Read File in the Time Course Data subpanel (upper left side of the main screen). Upon selection, the data will be validated by the GUI, which will promptly notify the user of any problem. If the data in the file is properly structured, the contents will be displayed in the Time Course Data subpanel text window. After reading the file, a plot of the temporal evolution of the species is made available by clicking Plot Data in the Time Course Data subpanel.

Using some *a priori* knowledge about the MM system, we set the input options to allow second order reactions and a maximum of one molecule for each species in every reaction (uni–molecular reactions). This is done using the Reaction Order and Molecularity subpanels of the Input panel, respectively. For illustrative purposes, we also chose to reconstruct the mechanism using two information criteria: Empirical and Akaike. The user can select one of three information criteria in the Information Criteria subpanel. Additionally, the user can choose a set of reactions to be excluded from the solution, using the Non-Possible Reactions subpanel of the Input panel. While we chose not to remove any reactions in this example, this feature will be used in the next example. We also fully describe the exclusion of unrealistic biochemical reactions in the Design and Implementation subsection (Methods).

By clicking the button Process in the Main panel, the GUI invokes MIKANA to predict a set of biochemical reactions for the designated input. If the computation is successful, the results are written in the text windows of the Output panel. To satisfy different users, the GUI provides different views of the same results. These views are accessed through the buttons Change View in the Used Reactions, Predicted Reactions, and Differential Equations subpanels of the Ouput panel.

When the Empirical information is selected, our method predicts 2 out of 44 elementary reactions. Together with the estimated kinetic rate constants, the two predicted reactions are governed by the following differential equations:
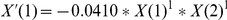


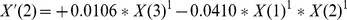


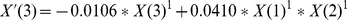


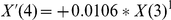



Here, 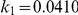, and 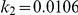. Note that MIKANA cannot reconstruct the reversible reaction 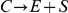 (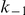 = 0). It is predicting the Van Slyke-Cullen reaction mechanism instead of the MM mechanism of enzyme action.

On the other hand, MIKANA can reconstruct correctly the MM reaction mechanism and estimate the kinetic parameters with the Akaike information criterion. The predicted differential equations are:
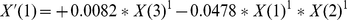


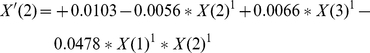


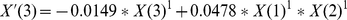


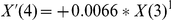



Here, 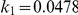, 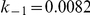 and 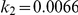. In this last example, 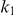, 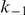 and 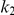 reveal differences of 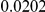 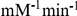, 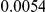 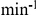 and 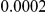 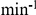, respectively, when compared to the original kinetic rate constants.

**Figure 3:**
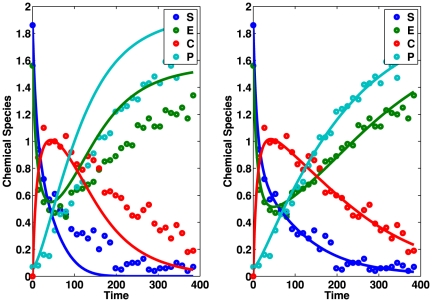
Input and Output time course data for the irreversible Michaelis–Menten reaction mechanism. The reaction mechanism describes interactions between four chemical species. Open circles represent the input time course data. Solid lines represent the output time course data. We produced the output using the Empirical (left plot) and the Akaike (right plot) information criteria. MIKANA cannot reconstruct the Michaelis–Menten reaction mechanism and its dynamical behavior using the Empirical information criterion. However, the reconstruction is excellent using the Akaike information criterion.

MIKANA can produce output time course data by solving the differential equations of the reconstructed mechanism. Figure 3 shows the output of MIKANA. Here, we show the input time course data (open circles) and the output course data (solid lines) using the Empirical (left) and the Akaike (right) information criterion. In the GUI, this plot is obtained by selecting the button Plot Input/Output Time Course in the main panel (lower right side of the main screen). The figure illustrates a difference in the reconstruction by using these two information criteria. Note that the Empirical information criterion provides a poor reconstruction; the fitting is poor and the mechanism is missing a reaction pathway. An estimate of the fitting error can be obtained by clicking Show Fit Errors in the main panel of the GUI. The user can also save all the results or just the output time course data to a file using the buttons Save Output to a File and Save Output Time Course to a File in the Main panel.

In this example, we considered second order reactions and a maximum of one molecule for each species in every reaction (uni–molecular interactions). However, the user can choose a different set of options for the mechanism reconstruction.

### Example 2 - The interaction map of chemical species of the muscle glycolytic pathway

Phosphocreatine (PCr) has been found to play an important role in the contraction of skeletal muscle cells [24]. During intense exercise, when the amount of oxygen available is lacking, PCr is broke down to creatine and its phosphate group (Pi). This phosphate group binds to ADP to form ATP, which is then used to contract muscles. Further, it is known that in intense exercise, PCr is rapidly depleted and does not provide enough ATP. The cell then uses another pathway as a source for ATP that leads to the production of lactate [21], [22], [25]. During the recovery period following the intense exercise, the ATP molecule produced by glycolysis is dephosphorylated, and the removed phosphate group added to creatine to replenish the phosphocreatine.

In an experiment by Scopes [21], lactate production and ATP synthesis by glycolytic flux was coupled to the creatine kinase flux so that the concentration of Pi decreased and the concentrations of PCr and lactate increased in the experimental mixture in vitro as a function of time. The mixture of substrates or products, reconstituted the muscle cell network function. We extracted time course data originally obtained by Scopes [21], [22]. These data comprise 14 time points measured over a period of 10 min for concentrations of PCr, Pi and Lactate. We interpolated these data to produce 3 time series of 26 points each over the same time period. The input time course data file for Scopes data consists of 4 columns: a time column with the time plus three columns with the chemical species time courses.

 Given that the experimental data does not contain a detailed measurement of all interacting species (enzymes and intermediates), we chose to proceed with the mechanism reconstruction assuming that the reactions follow a pseudo-first order kinetics. This will permit us to reconstruct the interaction map between the three chemical species. We chose to use a maximum of two molecules for each species in every possible reaction and an Empirical information criterion. We further tell MIKANA to exclude reactions having either PCr as a reactant and lactate as a product, or lactate as a reactant and PCr as a product. For the purpose, we use the following rules: 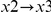 and 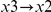. Here, x2 represents PCr and and x3 represents lactate (third and fourth columns of the time course data file, respectively). These rules were written to a file, which was loaded into the MIKANA GUI using the Non-Possible Reactions subpanel of the Input panel. The addition of these two rules produces 22 potential reactions and eliminates direct dependencies between the PCr and lactate in the final result. Glycolysis leads to ATP and lactate production in muscle and results in the phosphorylation of creatine, which means that PCr and lactate are associated through the phosphate (Pi).

The reconstruction leads to 7 elementary reactions. These reactions, together with the estimated kinetic rate constants, are described by the following differential equations:
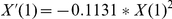


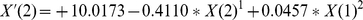


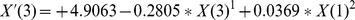



Even in the absence of a large number of unknown intermediates, MIKANA is capable of reconstructing a mechanism that agrees with the known relations between Pi, PCr and lactate. MIKANA predicts that Pi (X1) decreases independently of the concentrations of PCr (X2) and lactate (X3). Furthermore, MIKANA predicts that Pcr (X2) and Lactate (X3) growth inversely correlates with Pi (X1). When the concentration of Pi (X1) is high, Pi (X1) diminishes quickly while PCr (X2) and Lactate (X3) increase quickly. As Pi (X1) is depleted, both PCr (X2) and Lactate (X3) evolve to a steady state. Notice that PCr (X2) does not depend on lactate (X3) and vice-versa. They both only relate to the phosphate Pi (X1).

**Figure 4:**
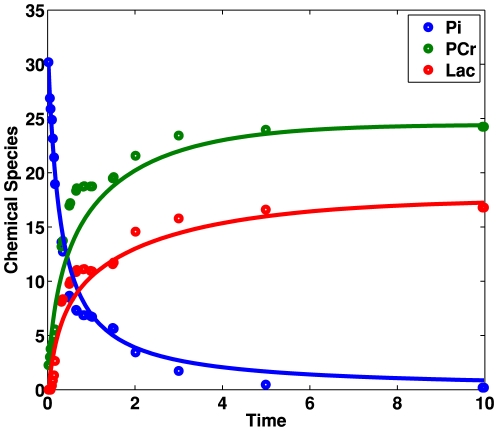
Input and Output time course data for chemical species of the muscle glycolytic pathway. The pathway describes interactions between phosphate (Pi), phosphocreatine (PCr) and lactate. Open circles represent the input time course data. Solid lines represent the output time course data. We produced the output using the Empirical information criteria. The predicted time course data provides a good qualitative fit to the experimental data. MIKANA also reconstructs the interaction relationships between Pi, PCr and lactate in the muscle cell pathway.

Figure 4 compares the experimental/input (open circles) and the predicted/output time course data (solid lines). The predicted time course data provides a good qualitative fit to the experimental data. The reconstruction of the relations between Pi, PCr and lactate without the removal of the direct relations between PCr and lactate produce 30 potential reactions and 6 elementary reactions as a result. However, this result includes direct relations between PCr and lactate that do not exist in the muscle cell pathway.

### Example 3 - The *Lactococcus lactis* Glycolytic pathway

The *Lactococcus lactis* glycolytic pathway has been explored experimentally in great detail [26], [27]. The glycolytic pathway converts glucose to lactate through a series of reaction steps. A simplified experimentally known topology involves the following steps: first, glucose (X1) is converted into glucose-6-phosphate (G6P) (X2). Phosphoenolpyruvate (PEP) (X5) also contributes to this step. G6P is converted into fructose-1,6-bisphosphate (FBP) (X3), then sequentially to glyceraldehyde-3-phosphate (Ga3P), 3-phosphoglyceric acid (3-PGA) (X4) and PEP (X5). Glucose and G6P, along with PEP, are involved in the conversion of PEP to pyruvate (X6). This step is activated by a positive feedback from FBP, which also exerts a positive feedback on the conversion of pyruvate to lactate (X7) [18].

 In this example, we used data from 13C (Nuclear Magnetic Resonance) NMR experiments [26]. These data comprise 25 time points measured over a period of 15.75 min, at an average time interval of half a minute. The input time course data file for the glycolytic pathway consists of a header line followed by numeric data. The header line contains 7 symbols and the numeric data consists of 25 lines and eight columns: a time column plus seven columns with the chemical species time courses. Given that the experimental data does not contain a detailed measurement of all interacting species (enzymes and intermediates), we chose to proceed with the mechanism reconstruction assuming that the reactions follow a pseudo-first order kinetics. We chose to use a maximum of two molecules for each species in every possible reaction and an Empirical information criterion. We do not exclude any potential reactions in MIKANA, rather we let it search through all possible first order reactions. The dictionary of elementary function produces 182 potential reactions. The reconstruction leads to 18 elementary reactions. These reactions, together with the estimated kinetic rate constants, are described by the following differential equations:
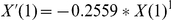


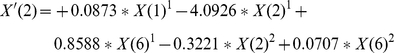


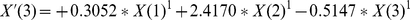


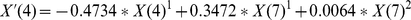


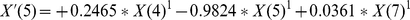


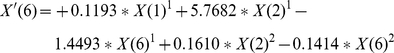


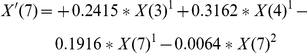



**Figure 5:**
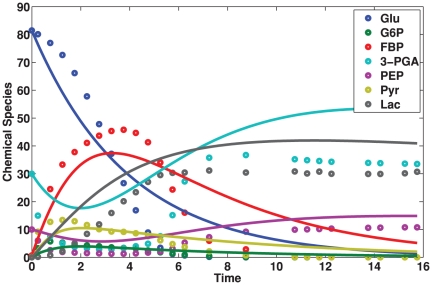
Input and Output time course data for the glycolytic pathway. The pathway describes interactions between seven chemical species. Open circles represent the input time course data. Solid lines represent the output time course data. We produced the output using the Empirical information criterion. The predicted time course data does not fit perfectly the experimental data. However there is a good qualitative agreement with the experimental data behavior. In addition, MIKANA is capable of reconstructing an interaction mechanism in close agreement with the *Lactococcus lactis* glycolytic pathway.

MIKANA is capable of reconstructing a mechanism in close agreement with the *Lactococcus lactis* glycolytic pathway. There are some small discrepancies. For example, MIKANA predicts that G6P (X2) is produced independently by glucose (X1) and pyruvate (X6). However, in reality, glucose (X1) and PEP (X5) are involved in the production of G6P (X2). MIKANA also predicts that FBP (X3) is produced by glucose (X1) and G6P (X2). Experimentally, glucose (X1) is not known to participate in FBP production. These discrepancies can be resolved by reconstructing the mechanisms with experimental replicates or under different initial conditions as we have shown before [19]. In Figure 5 we compare the experimental/input (open circles) and the predicted/output time course data (solid lines). The predicted time course data does not fit perfectly the experimental data because the reconstruction is pseudo-first order. However, it is in a good qualitative agreement with the experimental data.

## Discussion

MIKANA is a computational method to infer reaction mechanisms and estimate kinetic parameters of biochemical pathways from time course data. This paper presents a Graphical User Interface (GUI) for MIKANA. MIKANA's input consists of chemical species time course data and a set of reactions to be excluded from the inference. The user can select between three different types of information criteria: (i) Empirical; (ii) Akaike; and (iii) Bayesian, along with a choice for first– or second–order reactions (Reaction Order), and interaction molecularity (one or two molecules per chemical species per reaction). The output includes a set of reactions used in the model selection process and a set of differential equations generated from the inferred reactions and kinetic parameters. Furthermore, the user can visualize and compare the input and the output time course data produced by the method.

We illustrate the capacity of the GUI using three different examples. In the first example, we reconstruct the irreversible single-enzyme, single-substrate Michaelis–Menten reaction mechanism. In the second example, we reconstruct the interaction map of chemical species of the muscle cell pathway. In the third example, we reconstruct the metabolic pathway of the glycolytic pathway of *Lactococcus lactis*. As evidenced by our examples, a number of different options is available for the reconstruction of a reaction mechanism. The choice between these options should be made according to the background knowledge in each case. For example, if no a priori knowledge is available for the order and/or molecularity of the biochemical reactions, the user should consider all possible reaction steps for the known reactants. If the user knows the order and/or molecularity, the user can use the GUI to introduce this knowledge and exclude some of the possible reaction steps.

The GUI provides easy access to experimentalists or researchers alike to infer relations among chemical species. Hopefully, these predictions should aid in generating hypothesis that can be experimentally tested. In addition to the GUI, this paper presents a description of MIKANA's code and methods. The description we provide is in sufficient detail to allow researchers to further develop the code or reproduce the experiments described. By making MIKANA's GUI and code available to the scientific community, we hope to increase current knowledge on chemical species interactions, possibly foster working collaborations and expand the knowledge in the field.

A significant feature of MIKANA is that it uses a nonlinear modeling technique based on the law of mass action: the rate of any given elementary reaction is proportional to the product of the concentration reactant species. This technique produces plausible chemical reaction steps of a pathway [17]. MIKANA also infers both reaction mechanisms and their kinetic parameters.

MIKANA's performance is dependent on several quantitative and qualitative properties of the time course data. This information is important to experimentalists as it could allow them to setup their experiments in ways that optimize the network reconstruction. As described in a previous paper, we performed an extensive test using several combinations of parameter values and determined the properties that provide the best results. Among the data tested, the best results are obtained using the Empirical information criteria. We also tested different parameters such as initial chemical concentrations, experiments (time courses), number of data points and noise. For enzyme catalyzed reactions, we found optimal results when substrate and enzyme have roughly the same initial concentrations, when we used more than 3 time courses and more than 30 data points. We also found optimal results when noise is lower than 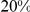 [19].

To the extent of our knowledge, there are not similar approaches available in the literature from which we can compare results. One possible source of comparison comes from the work of Voit [18]. His approach to modeling reactions is based on a power law approximation, proposed by Savageau [28], [29]: the power-law approximation assumes that the rate of change of a state variable is equal to the difference of two products of variables raised to non-integer powers. Voit [18] has used the power law approximation to study the regulation of glycolysis in the *Lactococcus lactis*, but the difference between this and MIKANA's approach makes it hard to compare results. In addition to the seven variables (each one represents a chemical species), Voit also decided to include as constants in his model other metabolites, such as ATP and NAD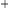. This makes any possible comparison inadequate.

### Availability and Future Directions

MIKANA is implemented in MATLAB (MATrix LABoratory). It is open-source and freely available for non-commercial and commercial use. The source code is available in Information S1. A standalone compiled version of MIKANA is also available for download at: http://sitemaker.umich.edu/schnell.lab/products. This website provides a quick guide for installing and using MIKANA. We also made available for download the three input time course data used as examples in this article. In this way, we have provided all the information necessary for anyone to replicate our results. In MIKANA, we traverse the search space of possible models by removing one reaction at a time from the initial set of all possible elementary reactions that compose the model. While possibly leading to better results, an exhaustive search would be impractical. To overcome this problem and potentially find better solutions, we are currently exploring alternatives to the one–reaction–at–a–time approach. One such alternative is to consider an heuristic method which would speed up the process of solution finding. At the moment, MIKANA also infers reaction mechanisms with the same or less number of chemical species than the ones used as input. Based on the current employed methods, we are analyzing the possibility of identifying unknown chemical species in the pathway. These and other limitations may be addressed by us or anyone else interested in our method. This would definitely result in possible extensions of MIKANA and the GUI.

## Methods

**Figure 6:**
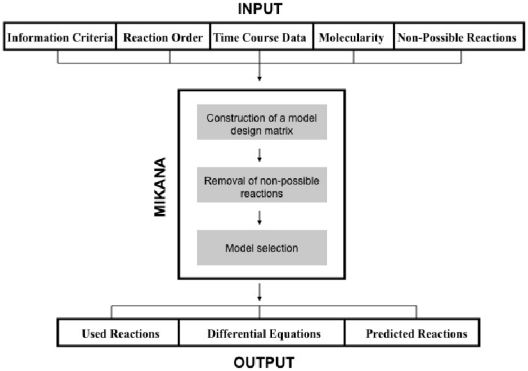
Input, Output and the main processes of MIKANA. MIKANA's input is composed of a time course data and a set of reactions known to be absent in the final solution set. There are three different types of information criteria: (i) Empirical; (ii) Akaike; and (iii) Bayesian. The user can also select between first– or second–order reactions (Reaction Order) and a maximum molecularity (one or two molecules per chemical species per reaction) to work with. The core process of MIKANA is the construction of a model design matrix with all possible elementary chemical reactions. Other processes include the exclusion of specified reactions and a model selection process. The output of MIKANA consists of a set of elementary reactions used in the model selection process and a set of predicted reactions from which we obtain the differential equations.

The input, main process components and output of MIKANA are shown in Figure 6. The input consists of time course data for chemical species and a set of reactions to be excluded from the inferred scheme. Users can select between three different types of information criteria: (i) Empirical; (ii) Akaike; and (iii) Bayesian, along with a choice for first- or second- order reactions (Reaction order) and interaction molecularity. The method predicts the reaction mechanism as a set of kinetic equations describing the rates of change of each chemical species in the pathway.

**Figure 7:**
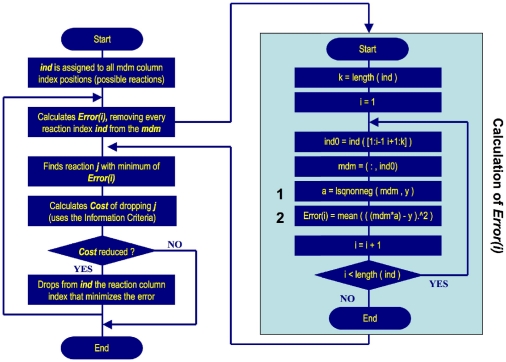
Model selection in MIKANA. The left flow chart shows the main steps taken in the model selection process. The right box details how we calculate the error of removing one reaction from the model design matrix mdm in MATLAB.

### MIKANA's main processes

The core process of MIKANA involves the generation of a complete dictionary of possible chemical interactions (which we will refer to as elementary reactions). A second process excludes specified reactions from the model design matrix. A final process applies a model selection technique to deduce the reaction mechanism. The latter process is aided by a cost function, an information criterion, that penalizes the use of an excessive number of reactions to reconstruct the pathway.

### Construction of a model design matrix

A key feature of our method lies in the construction of a model design matrix 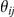 appropriate for biochemical pathways. The model design matrix is composed of columns representing unscaled velocities corresponding to all possible elementary reaction steps involving the different species in the pathway. Chemical reaction pathways are described by a number of elementary steps. Let us consider the general chemical elementary reaction:
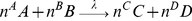
(2)Here, 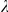 is the rate constant of the reaction and 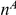, 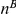, 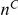 and 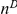 are the number of molecules (stoichiometry) of reactants 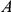 and 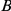, and products 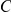 and 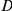. The velocity or rate of the above reaction is given, according to the law of mass action, by:
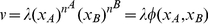
(3)where 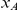 and 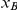 are the concentrations of species 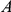 and 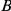, respectively. The above equation defines the unscaled velocity 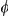, which describes the functional dependence of the reaction velocity on the concentration variables. Note that it does not depend on any further unknown parameters.

The above general framework is used to construct a set of chemically feasible elementary reactions if we restrict the reactions to a maximum molecularity. For example, for two species, the general elementary reaction can produce 18 chemically realistic schemes up to and including bi–molecular reactions:


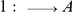
 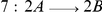 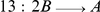



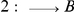
 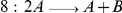 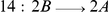



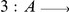
 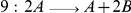 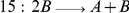



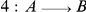
 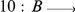 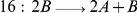



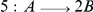
 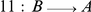 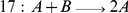



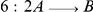
 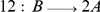 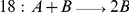


Here, 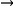 and 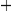 represent a reaction and an interaction between two different molecules, respectively. The general elementary set of chemical reactions is called the complete dictionary of basis functions or elementary reactions for two species [17].

For a species k, an element of the model design matrix is then defined as 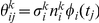. Here 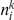 is the molecularity for species k in the 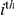 reaction, and the element 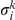 has unit magnitude with positive sign (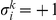) if k is a product and negative sign (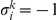) if k is a reactant for the 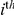 reaction. 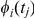 is the unscaled velocity for the 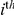 reaction, evaluated at the 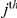 time point. The velocities are evaluated at each point in the time course and the model design matrix 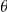 is constructed for each species as follows:
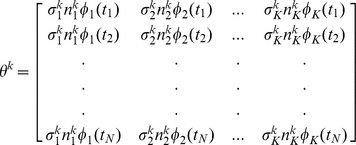



Only for those reactions in which species k takes part will n be nonzero. The overall matrix for the biochemical pathway is a concatenation of such matrices for each of the M species, resulting in a matrix of dimensions 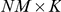, where N is the number of time points in the time course and K is the number of elementary reactions. A complete description of the construction of the model design matrix can be obtained in [17].

### Index and nindex matrices

In MATLAB, besides the model design matrix, we create two other auxiliary matrices, labeled index and nindex. Both matrices are useful throughout the program. Their size is 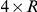, where 4 is the number of lines and R the number of columns or possible elementary reactions. While the first two lines refer to reaction reactants, the last two lines refer to reaction products. The index and the nindex matrices store the species identifier and the number of molecules that participate in the reaction, respectively. For example, the reaction 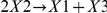 would add the following vectors to the index and nindex matrices:



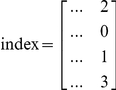


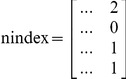


The construction of the index and the nindex matrices is essential to the process of exclusion of reactions in the final mechanism, as it is described below.

### Exclusion of biochemically unrealistic reactions

The number of possible elementary chemical reactions increases with the number of chemical species affecting the performance of MIKANA. We build a simple language that allows the user to introduce chemical species interaction information of biochemically unrealistic reactions. The language can also be used to identify known chemical interactions in the reaction. The syntax of our language makes the use of the following symbols:

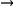
: Represents an irreversible reaction.

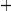
: Represents an interaction between two chemical species.

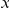
: Represents any generic chemical species.

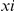
: Represents chemical species 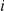 for 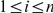. n stands for the number of chemical species for which a time course exists.

Examples of uni–molecular and bi–molecular interactions are, respectively: (i) 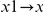; and (ii) 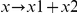. In a system with three chemical species, the first example would represent reactions 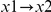 and 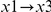.

We exclude reactions from the model design matrix (Figure 6), and from the index and nindex matrices, before applying the model selection process (Figure 6). The implementation of this section is mainly based on the outcome of evaluating the following expression:

[matchstr splitstr]  =  regexp(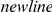, ‘[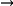 x +]’, ‘match’, ‘split’)

This expression searches for the symbols 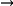, x and + in the string 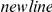. It further splits the string based on the matches found. The variable splitstr retains the number of molecules and the identifier of the chemical species that participate in the reaction. For example, if 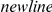 is 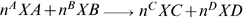, the variable splitstr is equal to: 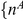 A 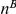 B 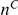 C 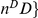. Such as for the index and nindex matrices described in the previous section, we add vectors with the splitstr outcome in two matrices of size 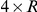, namely removal-index and removal-nindex. These matrices store the chemical species identifier and the number of molecules of each reaction identified, respectively. If the chemical species or the number of molecules is not specified, a value of 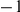 replaces the omitted values in the created vectors. The value 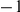 is used to represent any chemical species or any number of molecules. For example, the evaluation of the reaction strings: (i) 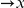; (ii) 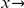; (iii) 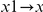; (iv) 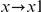; (v) 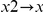; and (vi) 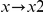, adds the following vectors to the removal-index and removal-nindex matrices:
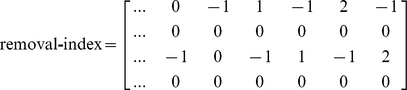


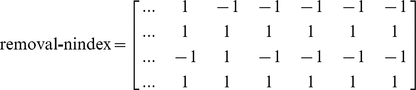



Here, the introduction of reactions (i) and (ii) could be used to define a closed system. Reaction (iii) would make the conversion of x1 into any other species without interacting with another chemical component impossible. Reversibly, reaction (iv) makes it impossible the conversion of any species into x1 without interacting with another chemical species. The last four reactions could be useful for introducing restrictions on chemical species interactions, such as to an enzyme or to a substrate, in an enzyme catalyzed reaction. Our next step is to identify which reactions in the matrices index and nindex match with the reactions in the matrices removal-index and removal-nindex. We exclude from the model design matrix and the matrices index, nindex all those reactions for which a match occurs. Those reactions are not considered in the model selection process (Figure 6), which we explain in the following subsection.

### Model selection

The iterative scheme proposed by Judd and Mees [30] uses a sensitivity analysis to determine the reaction which added or removed to a model, will most improve the model fit to the data. The model selection employed in MIKANA follows the latter approach (see [17] for more details). We show a flow chart of the most important steps of the model selection process in Figure 7. We start by assigning a vector ind with the column positions of all possible elementary reactions. We calculate a vector of errors where we assign to each position the error of removing that column reaction from the model design matrix. Using an information criterion, we calculate the cost of dropping the reaction that minimizes the error. If the cost of dropping this reaction is less than the previous cost (obtained with one more reaction), the cycle continues as we then attempt to remove another reaction. If the cost is not reduced, the cycle ends and the current reactions constitute the final mechanism.

**Errors.** To obtain the error for each reaction column position i (Figure 7, right side), we first obtain the non-negative least square solution of: (i) the model design matrix (mdm) without the 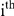 reaction; and (ii) the derivative vector y (step 1 in the box). Lsqnonneg returns the vector a that minimizes the norm(y-mdm 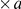) subject to 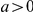. The norm of a vector V is defined as: sum(abs(V).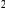 *)* 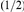. The derivative vector is obtained from the chemical species input time course data using the following equation:
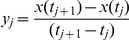



We then multiply mdm by the solution vector a and calculate the squared difference to the derivative vector y (step 2 in the box). The result is an error vector. Here, each point corresponds to an estimated error of using the current elementary reactions (columns of mdm) to explain a specific chemical species value on a time point.

**Cost.** To calculate the cost of dropping a reaction, we use an information criterion. The information criterion is used to penalize the use of an excessive number of reactions to reconstruct the pathway. We make three different types of information criteria available:
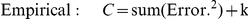


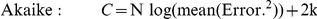


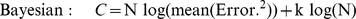



Here, Error is the error vector, N is the length of the Error vector and k is the number of reactions in the current model design matrix.

## Supporting Information

Information S1 **MIKANA Source Code and Examples.**
(ZIP)
